# Impact of contrast-enhanced transcranial Doppler ultrasound diagnosis for young adult with cryptogenic stroke: A protocol of systematic review

**DOI:** 10.1097/MD.0000000000018236

**Published:** 2019-12-16

**Authors:** Xiao-xue Jiang, You Song, Chun-rong Hu, Li-hua Wang, Lu Liu, Ya-juan Zhang

**Affiliations:** a Department of Neurology; b Department of Quality Control, First Affiliated Hospital of Jiamusi University, Jiamusi, China.

**Keywords:** contrast-enhanced transcranial Doppler ultrasound, cryptogenic stroke, sensitivity, specificity

## Abstract

**Background:**

This study aims to assess the impact of contrast-enhanced transcranial Doppler ultrasound (cTCD) diagnosis for young adult with cryptogenic stroke (CS).

**Methods:**

This study will analyze data from case-controlled studies investigating the impact of cTCD diagnosis for young adult with CS. A comprehensive literature search will be performed from PUBMED, EMBASE, Cochrane Library, Web of Science, Cumulative Index to Nursing and Allied Health Literature, Chinese Biomedical Literature Database, China National Knowledge Infrastructure, and Wanfang Data from their inceptions up to the August 1, 2019. All databases will be searched with no language limitations. Two researchers will independently carry out study selection, data collection, and study quality assessment. Any discrepancies between two researchers will be solved by a third researcher. We will apply RevMan 5.3 software and Stata 12.0 software for statistical analysis.

**Results:**

Outcomes consist of sensitivity, specificity, positive likelihood ratio, negative likelihood ratio, and diagnostic odds ratio for determination of cTCD diagnosis for young adult with CS.

**Conclusion:**

The results of this study may summarize up-to-date evidence of cTCD diagnosis for young adult with CS.

**Systematic review registration:**

PROSPERO CRD42019145641.

## 1 Introduction

Stroke is one of the most common neurological diseases, and it accounts for about 1 of every 20 deaths.^[1–4]^ Previous studies have reported that this condition is the fifth leading cause of mortality and also a major cause of morbidity among adult population.^[5–8]^ It has been estimated that about 795,000 stroke events attacking annually, and about 185,000 cases are recurrent ones in the United States.^[1]^ Of those, about 87% of them are ischemic strokes, and 25% to 39% of ischemic strokes have unknown cause, also known as cryptogenic stroke (CS).^[1,9]^ CS often occurs more common in young adults (<55 years of age).^[10–16]^ Therefore, extensive and rapid diagnostic is very important and necessary to help diagnosis CS.

Contrast-enhanced transcranial Doppler ultrasound (cTCD) diagnosis have reported to diagnosis patients with CS more effectively and accurately.^[17–25]^ However, it's results are still opposite, and no study has researched this topic at the evidence-based medicine level. Thus, this study will systematically assess the impact of cTCD diagnosis for young adult with CS.

## 2 Methods

### 2.1 Objective

This study will aim to investigate the impact of cTCD diagnosis for young adult with CS.

### 2.2 Eligibility criteria

#### 2.2.1 Type of studies

All case-controlled studies reporting the diagnostic accuracy of cTCD diagnosis for young adult with CS will be considered for inclusion in the final analysis.

#### 2.2.2 Type of participants

This study will include young adult participants (18–55 years old) with brain computed tomography or brain magnetic resonance imaging-proven CS, regardless their race and sex.

#### 2.2.3 Type of index test

Index test: We will utilize cTCD diagnosis for patients with CS.

Reference test: Patients with brain computed tomography or brain magnetic resonance imaging-proven CS will be used in the control group.

#### 2.2.4 Type of outcome measurements

Outcomes consist of sensitivity, specificity, positive likelihood ratio, negative likelihood ratio, and diagnostic odds ratio.

### 2.3 Data sources and search strategy

#### 2.3.1 Electronic searches

The main electronic databases of PUBMED, EMBASE, Cochrane Library, Web of Science, Cumulative Index to Nursing and Allied Health Literature, Chinese Biomedical Literature Database, China National Knowledge Infrastructure, and Wanfang Data will be assessed from their inceptions up to the August 1, 2019. All electronic databases will be presented with no language limitations. The search strategy for PUBMED is shown in Table 1. Similar search strategies will be adapted to other electronic databases.

**Table 1:**
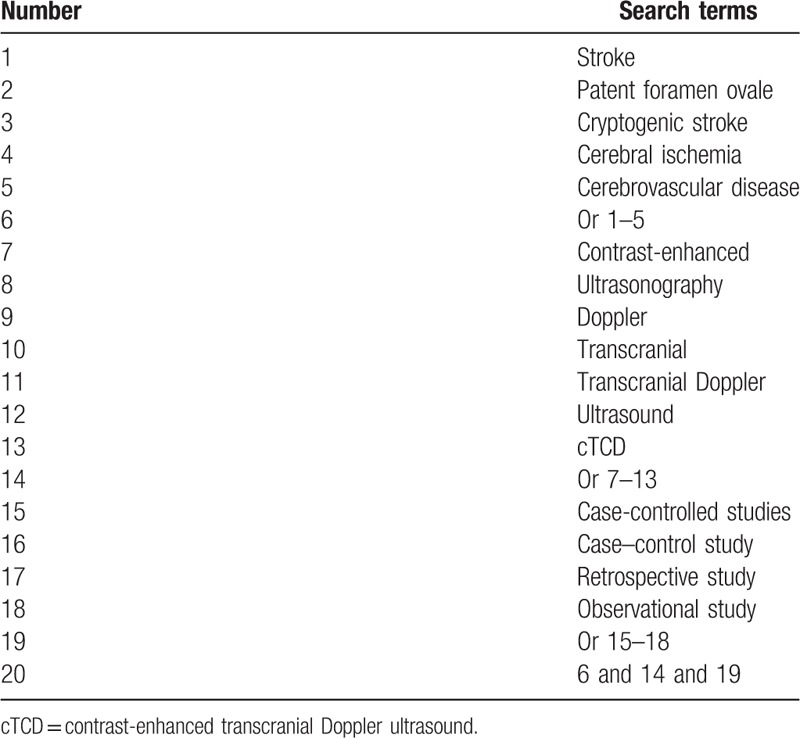
Search strategy for PUBMED.

#### 2.3.2 Other resources

Any relevant dissertations, Google scholar, and reference lists of associated reviews will be searched.

### 2.4 Data collection and analysis

#### 2.4.1 Selection of studies

According to the previous defined inclusion criteria, two researchers will independently check all literature records in all electronic databases. We will scan titles and abstracts of all searched studies; and irrelevant studies will be excluded. We will read full-text of all remaining studies, and these records will be retrieved for further evaluation to check if they meet all final inclusion criteria. Any disagreements regarding the study selection between two researchers will be solved by consensus with the help of a third researcher. The study selection process will be presented in the flow diagram.

#### 2.4.2 Data collection process

Two researchers will independently collect the data of all included studies according to the previous designed and standardized sheet. Any disagreements between two researchers will be solved by consensus with the help of a third researcher. We will extract the following data of trial characteristics (first author, time of publication, country, etc), patient characteristics (age, gender, race, etc), study design, study methods, details of diagnostic indexes, outcomes, including number of true positives and negatives, false positives and negatives, etc.

#### 2.4.3 Dealing with missing data

We will contact original authors of primary studies via email to inquire missing or insufficient, or unclear data if we identity the missing information during the period of data extraction. If we cannot obtain that information, we will analyze the available data and will discuss the potential impacts of such kind of data.

### 2.5 Assessment of methodological quality

Two researchers will independently assess the methodological quality for all eligible studies using Quality Assessment of Diagnostic Accuracy Studies.^[26]^ Any different opinions between two researchers will be solved by a third researcher via discussion. This tool has four fields, and each one is reported as risk of bias in each category.

### 2.6 Assessment of heterogeneity

The heterogeneity among included studies will be assessed by the *I*^*2*^ statistic test. Acceptable heterogeneity will considered if *I*^*2*^ ≤ 50%, while substantial heterogeneity will be regarded if *I*^*2*^ > 50%.

### 2.7 Subgroup analysis

Subgroup analysis will be performed to check the possible factors of significant heterogeneity based on the different types of characteristics of study and patient, indexes, and outcomes.

### 2.8 Sensitivity analysis

Sensitivity analysis will be carried out by eliminating studies with high risk of bias to check the stability and robustness of pooled results.

### 2.9 Reporting bias

If sufficient eligible studies are included in this study, a funnel plot will be conducted to check any possible publication bias.^[27]^

### 2.10 Statistical analysis

RevMan 5.3 software and Stata 12.0 software will be used to analyze the data and to pool the data if necessary. Outcome data will be expressed as descriptive statistics and 95% confidence intervals. We will utilize *I*^*2*^ statistic to identify the degree of statistical heterogeneity among eligible studies. *I*^*2*^ ≤ 50% exerts low heterogeneity, and a fixed-effect model will be used. If sufficient data is collected, we will carry out meta-analysis. *I*^*2*^ > 50% demonstrates obvious heterogeneity, and random-effect model will be utilized. In addition, we will perform subgroup analysis to check the possible reasons that result in significant heterogeneity. If there is still significant heterogeneity after subgroup analysis, we will report outcome results as narrative description.

### 2.11 Ethics and dissemination

This study does not require research ethic, because it will not analyze individual patient data. The results of this study are expected to be published on peer-reviewed journals.

## 3 Discussion

CS is one of the most common types of stroke. It often occurs without clear causes. It often attacks people < 55 years old, and greatly affects their quality of life. Therefore, quickly diagnosis for such disorder is very important. Previous studies have reported that cTCD diagnosis can be used for young adult with CS. However, no study has explored its impact for patients with CS. Thus, this study will systematically check the impact and accuracy of cTCD diagnosis for young adult with CS. Its results will summarize the up-to-date evidence of cTCD diagnosis for young adult with CS. Its findings may provide helpful reference for both clinical practice and future researches.

## Acknowledgments

This study was supported by Heilongjiang Provincial Health and Family Planning Commission Research Project (2018144). The financial support institute was not allowed to involve any sections of this study.

## Author contributions

**Conceptualization:** Xiao-xue Jiang, Chun-rong Hu, Li-hua Wang, Lu Liu, Ya-juan Zhang.

**Data curation:** Xiao-xue Jiang, You Song, Chun-rong Hu, Li-hua Wang, Lu Liu, Ya-juan Zhang.

**Formal analysis:** Xiao-xue Jiang, You Song, Chun-rong Hu, Li-hua Wang, Lu Liu.

**Investigation:** Ya-juan Zhang.

**Methodology:** Xiao-xue Jiang, You Song, Chun-rong Hu, Li-hua Wang.

**Project administration:** Ya-juan Zhang.

**Resources:** Xiao-xue Jiang, You Song, Chun-rong Hu, Li-hua Wang, Lu Liu.

**Software:** Xiao-xue Jiang, You Song, Chun-rong Hu, Li-hua Wang, Lu Liu.

**Supervision:** Xiao-xue Jiang, Ya-juan Zhang.

**Validation:** Xiao-xue Jiang, Chun-rong Hu, Li-hua Wang, Lu Liu, Ya-juan Zhang.

**Visualization:** You Song, Chun-rong Hu, Lu Liu, Ya-juan Zhang.

**Writing – original draft:** Xiao-xue Jiang, You Song, Chun-rong Hu, Li-hua Wang, Lu Liu, Ya-juan Zhang.

**Writing – review & editing:** Xiao-xue Jiang, You Song, Chun-rong Hu, Li-hua Wang, Lu Liu, Ya-juan Zhang.
